# Systematic comparation of the biological and transcriptomic landscapes of human amniotic mesenchymal stem cells under serum-containing and serum-free conditions

**DOI:** 10.1186/s13287-022-03179-2

**Published:** 2022-10-04

**Authors:** Yunyan Sun, Ti-er Wang, Qianwen Hu, Wenxia Zhang, Yun Zeng, Xun Lai, Leisheng Zhang, Mingxia Shi

**Affiliations:** 1 Department of Hematology, The First Affiliated Hospital of Kunming Medical University, Hematology Research Center of Yunnan Province, Kunming, 650032 China; 2 Department of Hematology, Yunnan Cancer Hospital, The Third Affiliated Hospital of Kunming Medical University, Yunnan Cancer Center, Kunming, 650118 China; 3 Department of Hematology, The Second Hospital of Hebei Medical University, Shijiazhuang, 050000 China; 4 Key Laboratory of Molecular Diagnostics and Precision Medicine for Surgical Oncology in Gansu Province & NHC Key Laboratory of Diagnosis and Therapy of Gastrointestinal Tumor, Gansu Provincial Hospital, Lanzhou, 730000 China; 5 Key Laboratory of Radiation Technology and Biophysics, Hefei Institute of Physical Science, Chinese Academy of Sciences, 350 Shushanhu Road, Shushan District, Hefei, 230031 Anhui China; 6 Center for Cellular Therapies, The First Affiliated Hospital of Shandong First Medical University, Jinan, 250014 China

**Keywords:** Human amniotic mesenchymal stem cells (hAMSCs), Serum-containing, Serum-free, Biological signatures, Transcriptomic characteristics

## Abstract

**Background:**

Human amniotic mesenchymal stem cells (hAMSCs) are splendid cell sources for clinical application in the administration of numerous refractory and relapse diseases. Despite the preferable prospect of serum-free (SF) condition for cell product standardization and pathogenic contamination remission, yet the systematic and detailed impact upon hAMSCs at both cellular and transcriptomic levels is largely obscure.

**Methods:**

For the purpose, we preconditioned hAMSCs under serum-containing (SC) and SF medium for 48 h and compared the biological signatures and biofunctions from the view of cell morphology, immunophenotypes, multi-lineage differentiation in vitro, cell vitality, cytokine expression, and immunosuppressive effect upon the subpopulations of T lymphocytes, together with the PI3K-AKT-mTOR signaling reactivation upon cell vitality. Meanwhile, we took advantage of RNA-SEQ and bioinformatic analyses to verify the gene expression profiling and genetic variation spectrum in the indicated hAMSCs.

**Results:**

Compared with those maintained in SC medium, hAMSCs pretreated in SF conditions manifested conservation in cell morphology, immunophenotypes, adipogenic differentiation, and immunosuppressive effect upon the proliferation and activation of most of the T cell subpopulations, but with evaluated cytokine expression (e.g., TGF-β1, IDO1, NOS2) and declined osteogenic differentiation and cell proliferation as well as proapoptotic and apoptotic cells. The declined proliferation in the SF group was efficiently rescued by PI3K-AKT-mTOR signaling reactivation. Notably, hAMSCs cultured in SF and SC conditions revealed similarities in gene expression profiling and variations in genetic mutation at the transcriptome level. Instead, based on the differentially expressed genes and variable shear event analyses, we found those genes were mainly involved in DNA synthesis-, protein metabolism-, and cell vitality-associated biological processes and signaling pathways (e.g., P53, KRAS, PI3K-Akt-mTOR).

**Conclusions:**

Collectively, our data revealed the multifaceted cellular and molecular properties of hAMSCs under SC and SF conditions, which suggested the feasibility of serum-free culture for the preferable preparation of standardized cell products for hAMSC drug development and clinical application.

**Supplementary Information:**

The online version contains supplementary material available at 10.1186/s13287-022-03179-2.

## Background

Mesenchymal stem/stromal cells (MSCs), also known as medicinal signaling cells, are heterogeneous cell populations with multi-lineage differentiation potential as well as unique hematopoietic-supporting and immunomodulatory properties [1, 2]. As the dominating stromal cells in the microenvironment, MSCs are orchestrated with other counterparts and mediate the self-renewal and differentiation of hematopoietic stem cells (HSCs) in physiological hematopoiesis and coordinate contribution to hematologic malignancies [3, 4]. To date, MSCs in combination with biomaterials or 3D-bioprinting scaffolds have been demonstrated with therapeutic effects in regenerative medicine by preclinical and clinical studies including aplastic anemia [5], acute myeloid leukemia (AML) [3, 6], B cell lymphoma [4], Crohn disease-associated enterocutaneous fistula [7], acute myocardial infarction (AMI) [8], premature ovarian failure (POF) [9–11], osteoarthritis [12], acute-on-chronic liver failure (ACLF) [13], Alzheimer’s disease [14], and even the coronavirus disease (COVID-19)-associated acute respiratory distress syndrome (ARDS) [15–17].

Since the first isolation from bone marrow in the 1960s, MSCs have been identified from various origins such as perinatal tissues (e.g., umbilical cord, amniotic membrane, placenta) [7, 18] and adult tissues (e.g., dental pulp of supernumerary teeth, adipose tissue) [19, 20] and even derived from pluripotent stem cells (e.g., ESCs, iPSCs) (Additional file 5: Table S1) [2, 12, 21]. Of them, hAMSCs have been acknowledged with multifaceted superiorities over other counterparts, and in particular, the preferable proliferation and immunomodulatory properties [22–24]. For instance, Navas et al. and Nasseri et al. have demonstrated the anti-fibrotic and anti-inflammatory effects of hAMSCs or conditioned medium in corneal and heart failure injury repair, respectively [25, 26]. However, most of the current references upon the characterization of the signatures are fragmentary and thus restrict the clinical application and drug development of hAMSCs-based cytotherapy. Of note, we and other investigators have reported the discrepancy of therapeutic effects of MSCs attributes to the variations in cell vitality, which is of great importance for standardizing clinical application of MSCs [27, 28].

In this study, we originally cultured hAMSCs in the aforementioned SC and SF medium to illuminate the possibility of harvesting standardized cell sources under serum-free condition to prevent contamination with pathogens and thus better satisfy the clinical demands and drug development purposes. For the purpose, we systematically compared the phenotypic characteristics and biological functions as well as transcriptomic signatures of hAMSCs under SC and SF conditions. Notably, compared with those in the SC group, hAMSCs maintained in SF medium revealed substantial conservation in cellular morphology, immunophenotypes, adipogenic differentiation, chromosome karyotype and inhibitory effects upon the subpopulations of cocultured T lymphocytes, together with comparable gene expression pattern and mutation spectrum. Interestingly, we also observed the reinforcement in most of cytokine expression in hAMSCs under SF precondition and the diversity in cell vitality including delayed cell cycle, decreased cell proliferation, and apoptosis. Collectively, our data suggested the feasibility of serum-free culture for large-scale and high-quality hAMSC preparation in future.

## Methods

### Subjects and hAMSC culture

The hAMSCs were isolated and identified from amniotic membrane of healthy donors with the maternal informed consent (negative for hepatitis, HIV, syphilis, and other serological reactions) and the approval of Ethics Committee of the First Affiliated Hospital of Kunming Medical University (approval number: KLL-2017-mxs) according to the guidelines of Helsinki Declaration. The amniotic membrane was repeatedly rinsed under aseptic conditions and then cut into small pieces and overlaid at the bottom of the culture flask supplemented with hAMSC culture medium as we reported [29]. HAMSCs at passage 3–8 were cultured in DMEM/F12 medium supplemented with or without 10% FBS (Australia), 10 ng/ml rhEGF (PeproTech, USA), 4 ng/ml rbFGF (PeproTech, USA), 1% NEAA and L-glutamine (Gibco, USA), 1% Penicillin and streptomycin (Thermo Fisher, USA) addition for 48 h. After reaching 80% fusion, hAMSCs were dissociated with 0.125% trypsin–EDTA (Gibco, USA) and collected by centrifugation at 300 × g for 5 min at room temperature (RT). Finally, hAMSCs were seeded and cultured at 37 ℃, 5% CO_2_ for further analyses.

### Flow cytometry (FCM) analyses

FCM analyses were conducted as we recently reported with several modifications [6]. In brief, 1 × 10^6^ cells at the indicated time points were washed and collected by 1 × PBS (Gibco, USA) and incubated with fluorescence-conjunct antibodies (PE-Cy7-CD73, FITC-CD90, APC-CD105, APC-CD44, PE-Cy7-CD11b, PE-Cy7-CD34, FITC-CD3, PE-Cy7-CD45, APC-HLA-DR, FITC-CD45RO, FITC-CD4, APC-CD4, APC-CD25, APC-Cy7-CD4, APC-IL-4, PE-Cy7-CD45RA, Percp-Cy5.5-IFN-γ, APC-Cy7-CD62L, PE-IL-17A, PE-Foxp3) in dark at 4 ℃ for 30 min. Then, the cells were washed with 1 × PBS and turned to FACS Canto II (BD Bioscience, USA) for detection and FlowJo 6.0 software (NIH, USA) for detection, and 2 × 10^4^ cells were gated and analyzed with FlowJo 6.0 software (NIH, USA). The antibodies are listed in Additional file 5: Table S2.

### Cell proliferation detection

The proliferation capacity of the indicated hAMSCs was detected by using the Cell Counting Kit-8 (CCK-8, Japan) and Ki-67 staining (BioLegend, USA)-based FCM assay. Briefly, hAMSCs were incubated with the CCK-8 reagents at 37 °C for 2 h. The absorbance at 450 nm was read by microplate reader (Thermo Fisher, USA).

### Cell apoptosis and cell cycle assessment

Cell apoptosis of hAMSCs under SC and SF conditions at the indicated time points was accomplished by utilizing the Annexin V and 7-AAD based FCM assay as we previously described [5]. In detail, hAMSCs under SC and SF conditions were collected and dyed with the commercial FITC Annexin V Apoptosis Detection Kit with 7-AAD (BioLegend, USA) according to the manufacturers’ instructions and detected by FCM assay (BD Biosciences, USA).

As to cell cycle analysis, hAMSCs under SC and SF conditions (preconditioned in SF medium for 48 h) were fixed with 70% cold ethanol (Tianjin, China) after washing with cold 1 × PBS (Gibco, USA). Then, the cells were incubated with the commercial PI/RNase staining solution (BD Pharmingen, USA) at 4 °C for 30 min and detected by using the FACS Canto II (BD Biosciences, USA).

### Adipogenic and osteogenic differentiation potential of hAMSCs

The multi-lineage differentiation potential of hAMSCs under SC and SF conditions toward adipocytes and osteoblasts was analyzed as we recently described [2, 7]. In detail, hAMSCs were cultured in SC medium for maintenance and passage, and then, the cells were, respectively, preconditioned with SC and SF conditions for 48 h, and then, the medium was changed into adipogenic and osteogenic differentiation medium (Stem Cell Technologies, USA) for two weeks. For adipogenic differentiation analysis, the hAMSC-derived adipocytes were washed and dyed with Oil Red O staining. For osteogenic differentiation analysis, the hAMSC-derived osteoblasts were dyed with Alizarin Red S staining. The morphology of the hAMSC-derived cells was recorded by using Nikon Eclipse Ti-U microscope (Nikon, Tokyo, Japan). The primer sequences of adipogenic and osteogenic differentiation-associated genes are listed in Additional file 5: Table S3.

### Tumorigenicity assay of hAMSCs

To evaluate the in vivo safety of hAMSCs preconditioned in SC and SF medium, we turned to tumor formation analysis as we previously reported [27]. Briefly, 1.0 × 10^7^ hAMSCs preconditioned in SC or SF medium for 48 h mixed with Matrigel were subcutaneously injected into nude mice (n = 3 in each group) for tumorigenicity assay. Mice with 1.0 × 10^6^ HeLa cells mixed with Matrigel injection were used as the positive control. After 12 weeks, the mice in the indicated groups were turned to H&E staining for tumor structure detection.

### Coculture of hAMSCs and T lymphocytes

To evaluate the immunoregulatory effects of hAMSCs pretreated with SF and SC (preconditioned in SF medium for 48 h) medium, we conducted coculture of the indicated hAMSCs and T lymphocytes as we previously described with several modifications [5, 6]. In detail, the CD3^+^ T cells were isolated and enriched from peripheral blood by using the Ficoll-Paque PLUS reagent (GE, Sweden) and magnetic activated cell sorting (MACS). Then, the T cells were stimulated with CD3 and CD28 antibodies-conjunct beads (BioLegend, USA) and cocultured with the aforementioned hAMSCs preconditioned in SC or SF medium in RPMI 1640 (Gibco, USA) basal medium supplemented with 10% FBS (Australia) and 1% Penicillin and streptomycin (Thermo Fisher, USA) for 48 h. Finally, the T cells were collected by centrifugation at 350 × g for 5 min and then labeled with fluorescence-conjunct antibodies for the quantitative analysis by FACS Canto II (BD Bioscience, USA) and FlowJo 6.0 software (NIH, USA).

### Quantitative real-time PCR (qRT-PCR) assay

Total RNAs of hAMSCs cultured in SC and SF (preconditioned in SF medium for 48 h) medium were harvested by using the TRIzol reagent (Thermo Fisher, USA) according to the manufacturers’ instructions. Then, cDNAs were synthesized by utilizing the Transcript Fly First-Strand cDNA Synthesis SuperMix (Transgen Biotech, China). After that, qRT-PCR analyses were performed with the primers (Shanghai Sangon Biotech, China), ABI PRISM 7900 (Applied Biosystems), and SYBR Green PCR Master Mix (Qiagen, Germany) as we described [2, 30]. The primer sequences are listed in Additional file 5: Table S3.

### Karyotypic analysis

The chromosome karyotyping analysis of hAMSCs preconditioned with SC and SF (preconditioned in SF medium for 48 h) medium was accomplished as we recently reported [2, 3]. In brief, we took advantage of the typical G-banding technique for the monitor of the genomic stability of the aforementioned hAMSCs [21]. The karyotype of hAMSCs was observed and recorded by using the Olympus DA71 microscope (Tokyo, Japan) under 200 × magnification.

### RNA-SEQ and bioinformatic analyses

The RNA-SEQ and bioinformatic analyses were conducted as we reported before [3, 5, 6]. Briefly, total mRNAs were isolated from hAMSCs cultured in SC and SF (preconditioned in SF medium for 48 h) conditions and then quantified by utilizing the NanoDrop 2000 (Thermo Fisher, USA). The RNA-SEQ was performed by Novogene (Tianjin, China) as we reported before [2, 30]. The bioinformatic analyses were conducted as we recently reported with the aid of the online databases and platforms [5, 12, 27]. The differentially expressed genes (DEGs) are listed in Additional file 6: Table S4. The genetic variations in hAMSCs under SC and SF conditions are listed in Additional file 7: Table S5.

### Statistical analysis

The statistical analyses were conducted with the Graph Pad Prism 6.0 (San Diego, USA) software as we reported before [31, 32]. The Student’s unpaired t test and the one-way ANOVA test were used for analyzing the data between two unpaired groups and among multiple unpaired groups, respectively. All data were represented as mean ± SEM and only when *P* value < 0.05 was considered statistically significant. NS, not significant; *, *P* < 0.05; **, *P* < 0.01; ***, *P* < 0.001; ****, *P* < 0.0001.

## Results

### hAMSCs under SC and SF conditions revealed similarity in immunophenotypes but moderate variation in osteogenic differentiation

**Fig. 1:**
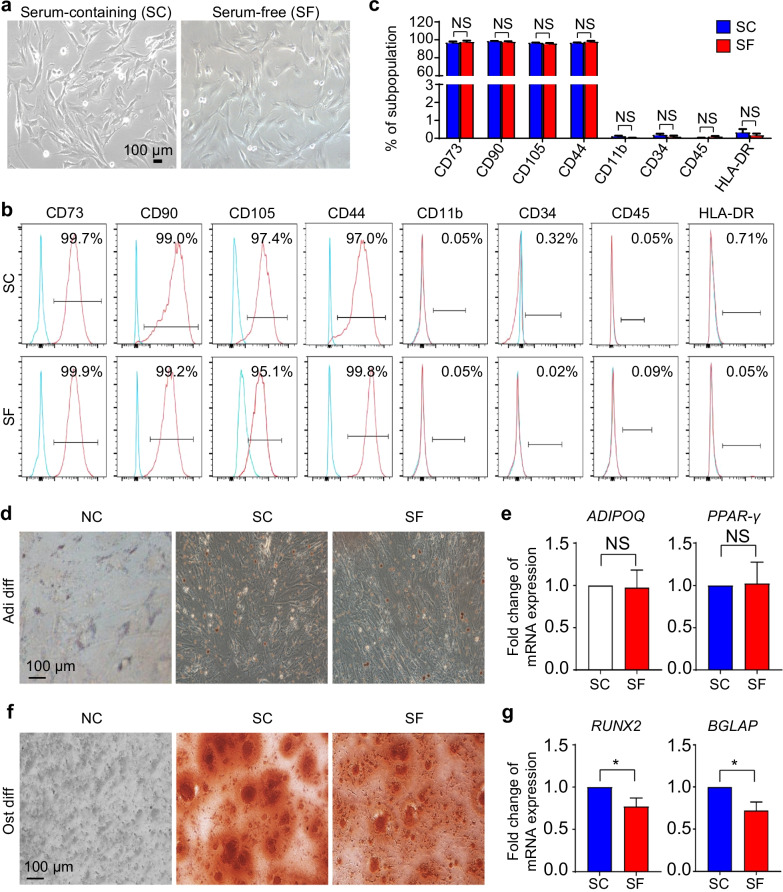
The identification of hAMSCs under serum-containing and serum-free conditions. **a** Representative phase contract images of hAMSCs under serum-containing (SC) and serum-free (SF) conditions. Scale bar = 100 μm. **b–c** Representative FCM diagrams **(b)** and statistical analysis **(c)** of surface markers of hAMSCs (2 × 10^4^ cells) in the indicated groups. All data were shown as mean ± SEM (*N* = 3). NS, not significant. **d** Representative images of hAMSC-derived adipocytes evaluated by Oil red O staining. NC, negative control. Scale bar = 100 μm. **e** Relative mRNA expression of adipogenic genes (*ADIPOQ*, *PPAR-γ*). **f** Representative images of hAMSC-derived osteoblasts evaluated by Alizarin Red S staining. NC, negative control. Scale bar = 100 μm. **g** Relative mRNA expression of osteogenic genes (*RUNX2*, *BGLAP*)

To dissect the potential impact of serum-free (SF) condition, we originally cultured hAMSCs under both serum-containing (SC) and SF conditions for 2 days. Both of the hAMSCs in the indicated groups revealed typical spindle-like morphology without obvious differences (Fig. 1a). According to the FCM analyses, we found hAMSCs in SF and SC conditions consistently with high level of MSC-associated surface biomarker (CD73, CD90, CD105, CD44) expression but with minimal hematopoietic-associated biomarker (CD11b, CD34, CD45) and HLA-DR expression (Fig. 1b–c). Furthermore, we turned to adipogenic and osteogenic differentiation to analyze the potential ex vivo multi-lineage differentiation properties. As shown by the Oil Red O staining, equal amounts of lipid droplets were generated from hAMSCs cultured in SC and SF conditions, which were further confirmed by qRT-PCR analysis of adipogenic-associated biomarkers (*ADIPOQ*, *PPAR-γ*) (Fig. 1d–e) *P* > 0.05). Dissimilarly, the osteogenic differentiation potential of hAMSCs pretreated under SF condition was weaker than that in the SC group as shown by Alizarin Red S staining and quantitative analysis of osteogenic-associated biomarkers (*RUNX2*, *BGLAP*) (Fig. 1f–g *P* < 0.05). Taken together, hAMSCs cultured in serum-free medium manifested conservation in cell morphology and immunophenotypes except for the moderate variation in osteogenic differentiation.

### The gene expression profiling of hAMSCs pretreated in SF and SC medium showed similarity and diversity

**Fig. 2:**
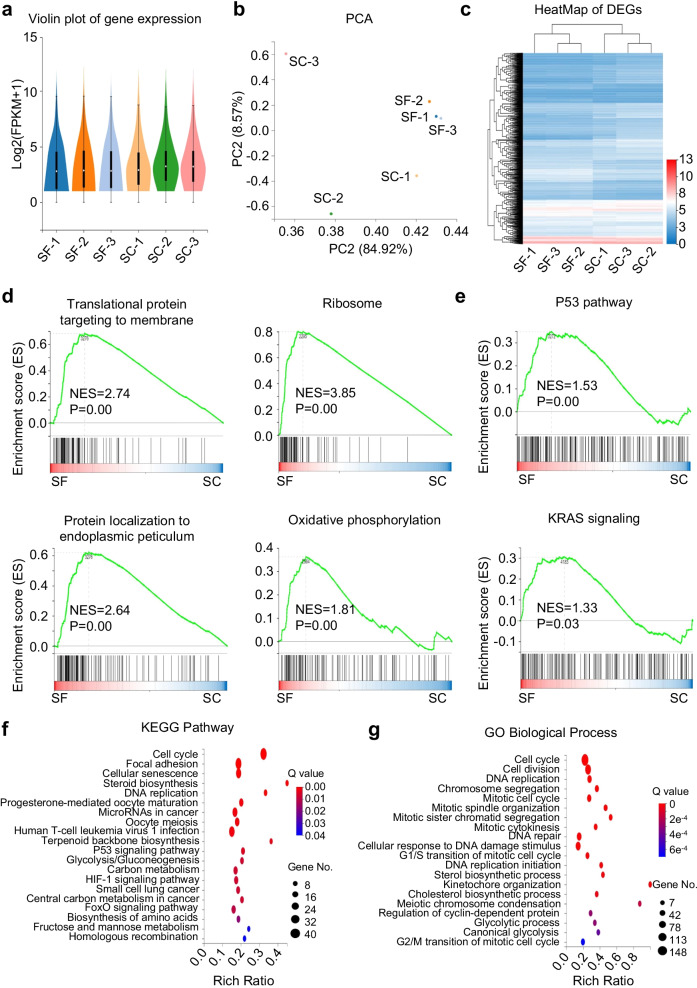
Comparation of gene expression profiling of hAMSCs in the SC and SF groups. **a** The violin plot of gene expression pattern in hAMSCs under SC and SF (preconditioned in SF medium for 48 h) conditions. **b** The PCA analysis of hAMSCs in the SC and SF groups based on FPKM values. **c** Heatmap diagram of differentially expressed genes (DEGs) in the indicated groups (SC and SF). **d-e** GSEA diagrams of hAMSCs under SC and SF conditions. **f-g** KEGG analysis **(f)** and gene otology biological process (GOBP) **(g)** of hAMSCs in the SC and SF groups based on DEGs

Having compared the cellular phenotypes of hAMSCs under SF and SC conditions, we next turned to dissect the signatures at molecular level. For the purpose, we took advantage of RNA-SEQ-based transcriptomic analyses and found that hAMSCs in the SF (SF-1, SF-2, SF-3) and SC (SC-1, SC-2, SC-3) groups exhibited similarity in gene expression distribution as shown by the fragments per kilobase per million (FPKM) value-based Violin Plots (Fig. 2a). Interestingly, according to the principal component analysis (PCA), hAMSCs cultured in SF medium revealed more clear and compact clustering when compared with those in the SC group (Fig. 2b). To dissect the detailed information of hAMSCs in the indicated groups, we further conducted heatmap analysis and intuitively observed the hierarchical cluster landscapes of differentially expressed genes (DEGs) (Fig. 2c).

To gain more insights into the potential influence of serum-free culture upon the transcriptomic signature of hAMSCs, we utilized the gene expression profiling-based Gene Set Enrichment Analysis (GSEA) and noticed that the DEGs were involved in multiple processes, and in particular, protein metabolism-associated processes such as translational protein targeting to membrane, protein localization to endoplasmic reticulum, ribosome, and oxidative phosphorylation (Fig. 2d, *P* < 0.01). Meanwhile, typical signaling pathways such as P53 and KRAS signal were also specifically enriched between the two groups (Fig. 2e, *P* < 0.055). Finally, with the aid of Kyoto Encyclopedia of Genes and Genomes (KEGG) pathway and gene ontology biological process (GOBP) analyses, we observed the potent influence of DEGs to hAMSCs under SC and SF conditions which were mainly abundant in DNA and protein metabolism (e.g., DNA replication and repair, steroid biosynthesis) as well as cell vitality-associated processes (e.g., cell cycle, cell division, G2/M transition of mitotic cell cycle) (Fig. 2f–g). Collectively, hAMSCs maintained in SF medium showed better clustering than those in the SC medium, and the DEGs between the two groups were mainly involved in cell vitality-associated signaling cascades and processes such as protein metabolism and DNA synthesis.

### The landscapes of genetic mutations and variation spectrum of hAMSCs preconditioned in SC and SF medium

**Fig. 3:**
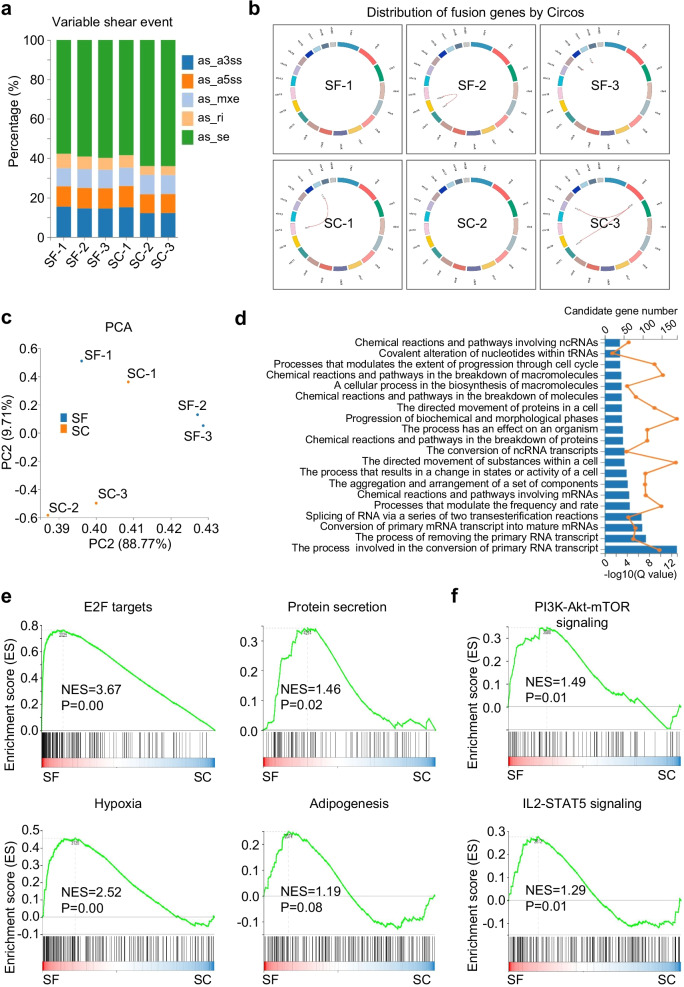
Variations of genetic mutations and variation spectrums in hAMSCs under SC and SF conditions. **a** Variable shear events in hAMSCs under SC and SF conditions. **b** Circos diagrams showed the distribution of fusion genes in hAMSCs under SC and SF conditions. **c** PCA diagrams revealed the distribution of hAMSCs in the indicated groups. **d** GOBP analysis of genes with genetic mutations and variation spectrums in hAMSCs under SC and SF conditions. **e–f** GSEA diagrams revealed the GOBP **(e)** and signaling pathways **(f)** in the indicated groups

To further disclose the genetic characteristics hAMSCs cultured in SF and SC medium, we compared the two groups from the perspectives of genetic modification. As shown by the diagram of variable shear events (VSEs), hAMSCs in the indicated two groups consistently with high percentages of as_se variation, moderate as_a3ss, as_a5ss and as_mxe variations but with minimal as_ri variation instead (Fig. 3a). From the overview of Circos diagrams, we did not observe regular tendency of fusion gene distribution between the two groups, which was further confirmed by the PCA diagram (Fig. 3b–c). According to the GOBP columns, the genetic mutations and variations were mainly enriched in multiple primary RNA transcripts (e.g., ncRNAs, tRNAs, mRNAs), biosynthesis, cell cycle and activity (Fig. 3d). Furthermore, as shown by the GSEA diagrams, the genetic modifications between the two groups were involved in metabolism-associated bioprocesses (e.g., E2F targets, protein secretion, and hypoxia) and signals associated with cell vitality and immunomodulation (e.g., PI3K-Akt-mTOR, IL2-STAT5) rather than adipogenesis (Fig. 3e–f). Taken together, hAMSCs maintained in serum-free condition exhibited similar genetic characteristics and genomic stability, and those variation spectrums were mainly involved in cell vitality-associated metabolism and immunomodulatory processes.

### hAMSCs maintained in SC and SF conditions showed multifaceted variations in cell vitality but with consistent safety in vivo

**Fig. 4:**
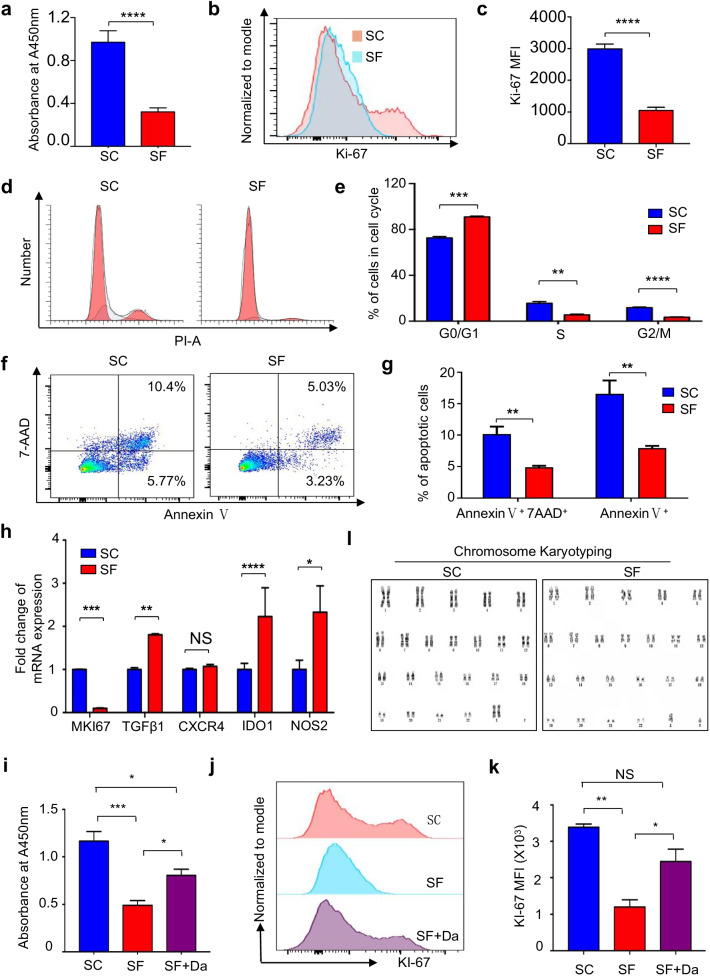
Comparation of cell vitality and cytokine secretion of hAMSCs under SC and SF conditions.** a** The proliferation of hAMSCs under SC and SF conditions. **b-c** Representative FCM diagrams **(b)** and statistical analysis **(c)** of Ki-67 MFI (mean fluorescence intensity) in hAMSCs under SC and SF conditions. **d-e** Representative FCM diagrams **(d)** and statistical analyses **(e)** of the distributions of the sub-stages of cell cycle in hAMSCs under SC and SF conditions. **f–g** Representative FCM diagrams **(f)** and statistical analyses **(g)** of the pre-apoptotic or apoptotic property of hAMSCs under SC and SF conditions. **h** Relative mRNA expression of the indicated cytokines in hAMSCs under SC and SF conditions. **i** The proliferation of hAMSCs under SC, SF, and SF + Da (10 nM 1,3-dicaffeoylquinic acid for PI3K-AKT-mTOR reactivation, denote as “Da”) conditions. **j–k** Representative FCM diagrams **(j)** and statistical analysis **(k)** of Ki-67 MFI in hAMSCs under SC, SF, and SF + Da conditions. **l** Representative G-banded chromosome karyotyping of hAMSCs under SC and SF conditions. Data were shown as mean ± SEM (N = 3). *, *P* < 0.05; **, *P* < 0.01; ***, *P* < 0.001; ****, *P* < 0.0001; NS, not significant

According to the transcriptomic analyses of DEGs and variation spectrums, hAMSCs cultured in SC and SF conditions indicated variations in cell vitality. To evaluate the potential influence of serum-free culture upon the cell vitality, we originally took advantage of the CCK-8 and Ki-67 analyses and found the significant decline in the proliferation of hAMSCs maintained in SF medium compared to the SC group (Fig. 4a–c). Similarly, distinguishing from the SC group, hAMSCs cultured in SF medium also showed decreased cell division according to the cell cycle phase distribution analysis (Fig. 4d–e). Interestingly, we found that the proportions of proapoptotic and apoptotic cell populations in the SF group were consistently reduced, whereas most of the cytokines were significantly upregulated in hAMSCs cultured in SF condition (Fig. 4f–h).

As mentioned above, based on the results of bioinformatic analyses of hAMSCs preconditioned in SC and SF medium for 48 h, we noticed the specific enrichment of PI3K-Akt-mTOR signaling pathway. To verify the potential role of this signals upon the cell vitality of the aforementioned hAMSCs, we took advantage of the activator of the signal (10 nM 1,3-dicaffeoylquinic acid, denoted as “Da”) and found that the reactivation of PI3K-Akt-mTOR signaling pathway in hAMSCs preconditioned in SF medium (SF + Da) was benefit for the considerable rescue of the declined proliferation rather than the apoptosis (Fig. 4i–k, Additional file 1: Figure S1a-S1b). Additionally, with the aid of G-banded chromosome karyotyping, we identified that hAMSCs in the SC and SF groups exhibited normal karyotype without the gross abnormalities at the chromosomal level (Fig. 4l). To evaluate the in vivo safety of the aforementioned hAMSCs, we took advantage of the subcutaneous tumorigenesis test as we previously reported [27] and found that there were no tumor structures formed in nude mice by hAMSCs preconditioned in SC or SF medium for 48 h (Additional file 1: Figure S1c-S1d). Collectively, in consistent with the transcriptomic prediction, hAMSCs maintained in serum-free medium manifested multifaceted diversity in cell vitality compared to those cultured in serum-containing circumstance but with consistent safety in vivo.

### hAMSCs preconditioned in SC and SF medium exhibited similarity in modulating the proliferation and activation of T lymphocyte subpopulations

**Fig. 5:**
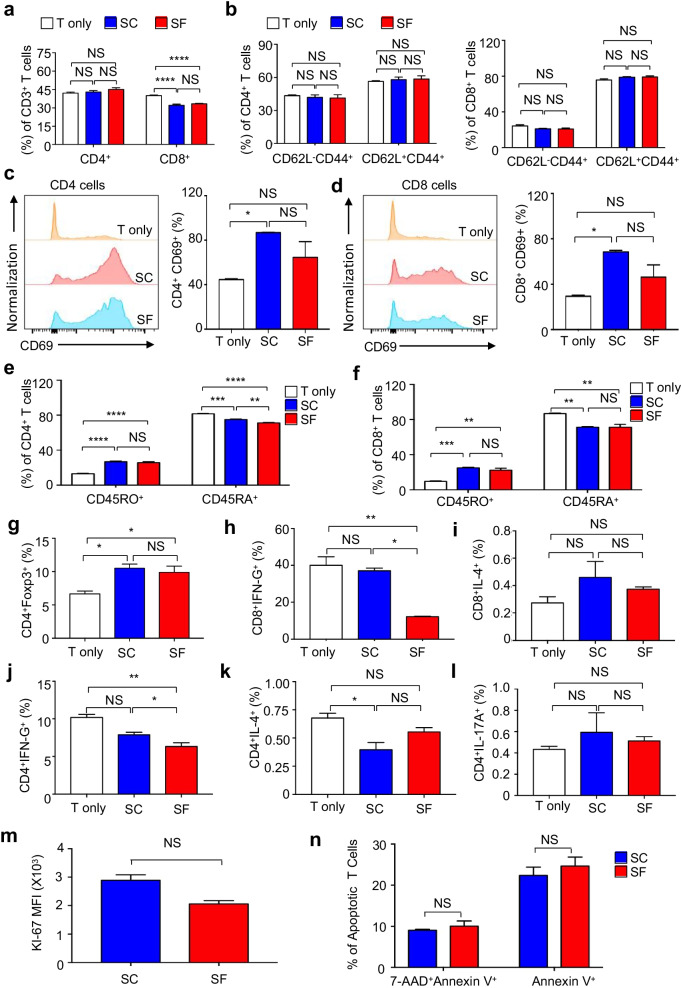
Comparation of the ex vivo immunoregulatory property of hAMSCs under SC and SF conditions. **a–b** The inhibitory effects of hAMSCs preconditioned in SC and SF conditions for 48 h upon CD3^+^ T cells (**a**), CD4^+^CD44^+^ T cells (left panel), CD8^+^CD44^+^ T cells (right panel) (**b**). **c–d** The representative diagrams (left panel) and statistical analyses (right panel) of CD4^+^CD69^+^ T cells (**c**) and CD8^+^CD69^+^ T cells (**d**) cocultured without (T only) or with hAMSCs preconditioned under SC and SF conditions. **e–f** The proportions of CD45RO^+^ and CD45RA^+^ subsets in CD4^+^ T cells (**e**) and CD8^+^ T cells (**f**) in the indicated groups. **g–i** The proportions of CD4^+^Foxp3^+^ Treg **(g)**, CD8^+^IFN-γ^+^ Tc1 (**h**), and CD8^+^IL-4^+^ Tc2 (**i**) subpopulations in the indicated groups. **j-l** The proportion of CD4^+^IFN-γ^+^ Th1 (**j**) and CD4^+^IL-4^+^ Th2 (**k**) and CD4^+^IL-17A^+^ Th17 (**l**) cells in the indicated groups. **m–n** Statistical analysis of the cell growth (**m**) and apoptosis (**n**) of the cocultured CD3^+^ T lymphocyte subpopulations with hAMSCs preconditioned in SC and SF conditions for 48 h. Data were shown as mean ± SEM (N = 3). *, *P* < 0.05; **, *P* < 0.01; ***, *P* < 0.001; ****, *P* < 0.0001; NS, not significant

Immunomodulation has been recognized as the unique and remarkable feature of MSCs, which is typically evaluated by coculturing with T lymphocytes as described in “Methods” section [5, 27]. To compare the immunosuppressive effects of hAMSCs in the SC and SF groups, we isolated CD3^+^ total T cells from peripheral blood by magnetic activated cell sorting (MACS) and cocultured with the aforementioned hAMSCs after irradiation for 48 h. As shown by the FCM diagrams and statistical analysis, hAMSCs pretreated in SC and SF conditions showed indiscriminate inhibitory effects upon the CD3^+^CD8^+^ cells, but with no obvious effects upon other subsets including CD3^+^CD4^+^, CD4^+^CD44^+^, and CD4^+^CD44^+^ T cell subsets (Fig. 5a–b, Additional file 2: Figure S2a-S2c). Consistently, both of the hAMSCs comparably enhanced the activation of CD4^+^CD69^+^ and CD8^+^CD69^+^ counterparts of the cocultured T cells (Fig. 5c–d). Moreover, the proportions of CD4^+^CDRO^+^ memory-like T cells were conformably increased accompanied with the decrease in the CD4^+^CD45RA^+^ and CD8^+^CD45RA^+^ native T cell counterparts by the cocultured hAMSCs, respectively (Fig. 5e–f, Additional file 3: Figure S3a). Similarly, hAMSCs pretreated with SF and SC conditions exhibited coordinate promoting effect to the CD4^+^Foxp3^+^ regulatory T cells (Tregs) and inhibitory action to the CD8^+^IFN-γ^+^ Tc1 cells but showed minimal impact upon the CD8^+^IL-4^+^ Tc2 cells (Fig. 5g–h, Additional file 3: Figure S3b). Finally, except for the preferable inhibitory action upon CD4^+^IFN-γ^+^ Th1 cells, hAMSCs preconditioned with SC and SF medium manifested equal repressive effects upon CD4^+^IL-4^+^ Th2 cells but revealed minimal impact upon the CD4^+^IL-17A^+^ Th17 cells and CD8^+^IL-17A^+^ T cells (Fig. 5i–l, Additional file 3: Figure S3c-3d). Additionally, with the aid of FCM analysis, we found that there were minimal differences in cell growth and apoptosis of T lymphocyte subpopulations when cocultured with hAMSCs preconditioned in SC and SF medium for 48 h (Fig. 5m–n, Additional file 4: Figure S4a-S4b).

## Discussion

A state-of-the-art renewal in preclinical studies and clinical practice has suggested the promising prospects of MSC-based cytotherapy in the administration of refractory and relapsing diseases [33, 34]. Distinguishing from the adult tissue-derived MSCs with limitations in proliferation and ethical risk, stem cells derived from the “discarded” amniotic membrane (hAMSCs) have been acknowledged as unlimited and quality-controllable alternatives with robust proliferative capacity for satisfying the large-scale clinical application and investigational new drug (IND) development [22, 35]. For decades, we and other investigators have devoted to illuminate the signatures and biofunctions of hAMSCs, yet the systematic characterization of the landscape of characteristics both at the cellular and molecular levels is largely obscure, which dramatically hinders the standardization of hAMSC-based remedies in regenerative medicine [26, 36]. Herein, we systematically elaborate the landscapes of biological phenotypes, transcriptomic signatures, and immunoregulatory effects upon T cell subsets of hAMSCs under serum-containing and serum-free conditions, which collectively supply overwhelming new references and will benefit the medical drug product development by utilizing the serum-free culture strategy without the risk of pathogen contamination.

The amniotic membrane is a “discarded” tissue of particular interesting after parturition, which contains epithelial and mesenchymal stem cells with low immunogenicity, robust proliferation, multi-lineage differentiation capacity, and easy procurement [37–39]. Therefore, hAMSC-based therapy has been widely explored to promote endogenous regeneration and alleviate the untoward effect of traditional treatment [40]. To date, hAMSCs have been reported as appropriate sources with therapeutic effects in regenerative medicine and tissue engineering such as premature ovarian insufficiency (POI) [41], premature ovarian failure (POF) [36], wound healing and epithelialization [42], hepatocellular carcinoma [43], and endogenous bone regeneration [40]. Generally, hAMSCs function via direct- and trans-differentiation, paracrine, and autocrine (e.g., extracellular proteins, anti-bacterial peptides, growth factors, angio-modulatory cytokines, bioactive factors), immunoregulation (e.g., anti-inflammatory agents, polarization of T lymphocytes, or macrophages), activating endogenous regeneration and serving as constitutive microenvironment [17, 39–41]. For instance, Li and the colleagues verified the alleviative apoptosis and intensive proliferation of skin cells during wound healing via activating PI3K/AKT signaling pathway, whereas He et al. recently put forward the therapeutic mechanism of hAMSCs via the LOXL2-mediated migration and differentiation of keratinocytes during wound epithelialization [38, 42]. Additionally, the amniotic membrane matrix with various types of extracellular proteins (e.g., laminins, collagens, fibronectins) can also serve as splendid anchors and ideal scaffolds or skin substitutes for cell attachment and proliferation and even sustained drug release [39].

Above all, the high-quality hAMSCs with standardization are the prerequisites of guarantee for disease treatment and IND development [35]. Herein, we systematically explored the potential influence of serum-free culture to the phenotypes and transcriptome of hAMSCs. Of note, Pianta et al. found that hAMSCs and the conditioned medium (CM-hAMSCs) were adequate to skew T cell polarization as well as downregulate the Th1 cell subsets by conducting the mixed lymphocyte coculture (MLC) [24]. Consistently, our data confirmed the preferable regulatory effects upon Th1 cell counterparts by hAMSCs under SF condition and further verified the comparable immunoregulation upon the proliferation and activation of T cell subsets by those cultured in SC medium. Our findings ultimately confirmed the feasibility of manufacturing large-scale and GMP-grade cell products under serum-free circumstances for clinical application and drug development purposes.

Nevertheless, more systematic and detailed analyses are urgently needed upon the multifaceted characteristics such as the multi-potent differentiation potential of hAMSCs toward hematopoietic, neural, and epithelial differentiation conditions in addition to osteogenic and adipogenic differentiation under SC and SF medium. Of note, the pioneering investigators in the field have also highlighted the methodology and feasibility of evaluating the biofunctions and underlying mechanisms of hAMSCs by comparing with relative sources of MSCs (e.g., BM-MSCs, P-MSCs) as well [44, 45]. Moreover, with the aid of transcriptomic analyses, we verified that the variations in DEGs and genetic modification were mainly involved in cell vitality-associated biological processes (e.g., protein secretion and localization, DNA synthesis, and replication) and signaling cascades (e.g., P53, PI3K-AKT-mTOR, KRAS). Additionally, based on the biological phenotypes and transcriptomic analyses, we also noticed the donor-to-donor variation. Therefore, it would be more interesting to see a motif enrichment analysis between the DEGs to see if there is an enrichment for genes with a serum-responsive element close to their promoter, enhancer, or TSS regions among the downregulated genes. Collectively, our data suggested the considerable conservation and comparable signatures of hAMSCs maintained in serum-free conditions compared to those cultured in serum condition, which will benefit the further explorations for hAMSC-based preclinical and clinical investigations and accelerate the application of hAMSCs in regenerative medicine.

## Conclusions

In this study, we conducted systematic characterization of the biological signatures and transcriptomic properties of hAMSCs cultured in serum-containing and serum-free medium. Notably, hAMSCs maintained in SF condition revealed considerable conservations both at the cellular and molecular levels, and the DEGs and genetic variations were mainly involved in cell vitality rather than other phenotypes including immunoregulatory effects upon the proliferation and activation of T lymphocytes. Collectively, our findings supply new references and will benefit the clinical application and IND development of hAMSC-based cytotherapy in regenerative medicine.

## Supplementary Information

**Additional file 1**: **Figure S1**. Cell vitality assay of hAMSCs with PI3K-AKT-mTOR signal reactivation and tumorigenicity assay.

**Additional file 2**. **Figure S2**: Comparation of the T cells after coculturing with or without hAMSCs pretreated in SC and SF conditions.

**Additional file 3**. **Figure S3**. Comparation of the inhibitory effects of hAMSCs pretreated in SC and SF conditions upon T cells.

**Additional file 4**. **Figure S4**. Cell growth and apoptosis of T lymphocyte subpopulations when cocultured with hAMSCs.

**Additional file 5**. The details accompanied with the main manuscript including Additional Figure Legends for Figure S1-S4, Additional Table S1-S3, Additional References were listed.

**Additional file 6**. **Table S4**. FPKM values of DEGs in hAMSCs under SC and SF conditions.

**Additional file 7**. **Table S5**. Genetic variations in hAMSCs under SC and SF conditions.

## Data Availability

All data of this study including the additional files are included in this published article. Meanwhile, the datasets involved in this study are available from the corresponding authors on reasonable request.

## Abbreviations

hAMSCs: Human amniotic mesenchymal stem cells
SF: Serum-free
SC: Serum-containing
DEGs: Differentially expressed genes
VSE: Variable shear event
MSCs: Mesenchymal stem/stromal cells
HSCs: Hematopoietic stem cells
AML: Acute myeloid leukemia
AMI: Acute myocardial infarction
PCA: Principal component analysis
POF: Premature ovarian failure
FPKM: Fragments per kilobase million
ACLF: Acute-on-chronic liver failure
COVID-19: Coronavirus disease 2019
ARDS: Acute respiratory distress syndrome
MACS: Magnetic activated cell sorting
MLC: Mixed lymphocyte coculture
GSEA: Gene Set Enrichment Analysis
KEGG: Kyoto Encyclopedia of Genes and Genomes
GOBP: Gene ontology biological process
POI: Premature ovarian insufficiency
IND: Investigational new drug
