# Supplementary figures and images for: Systematic comparation of the biological and transcriptomic landscapes of human amniotic mesenchymal stem cells under serum-containing and serum-free conditions
Source: Stem Cell Res Ther. 2022 Oct 4;13:490. doi: 10.1186/s13287-022-03179-2 (PMC9530421; doi:10.1186/s13287-022-03179-2)

**a**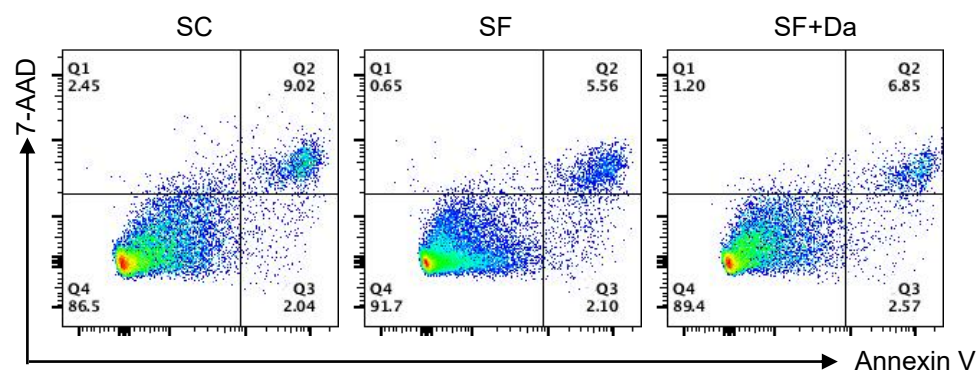**b**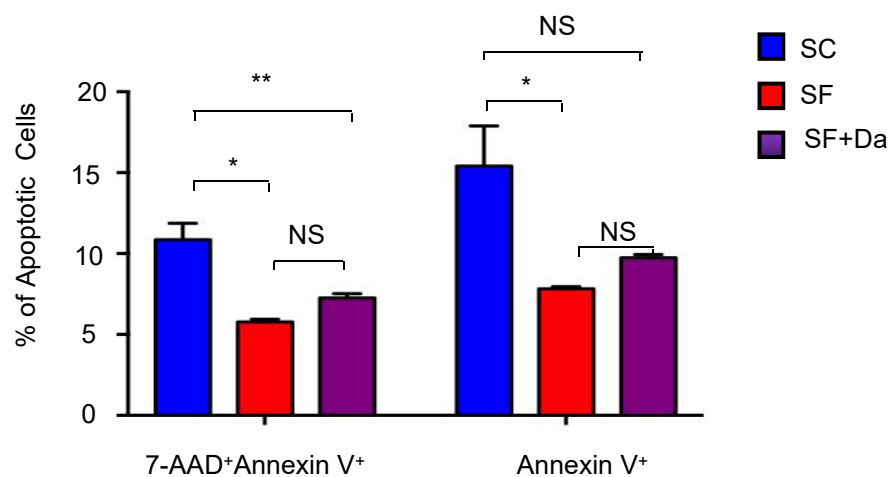**c**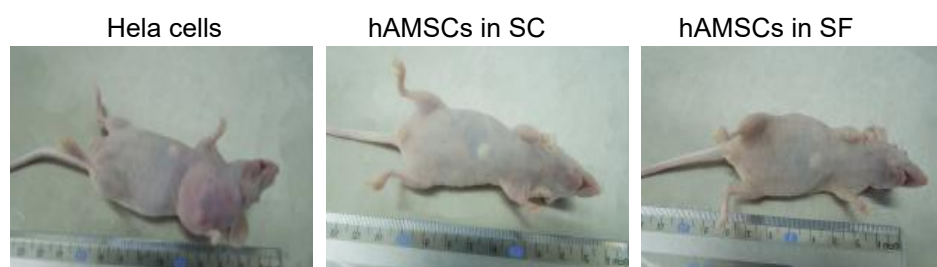**d**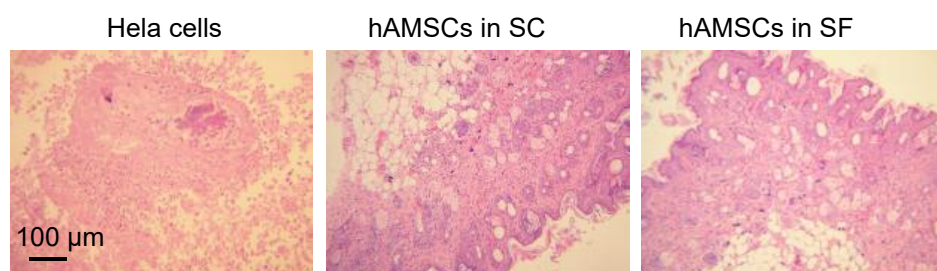

Supplement: Supplementary file 1 — Additional file 1: Figure S1. Cell vitality assay of hAMSCs with PI3K-AKT-mTOR signal reactivation and tumorigenicity assay. [file 13287_2022_3179_MOESM1_ESM.pdf]

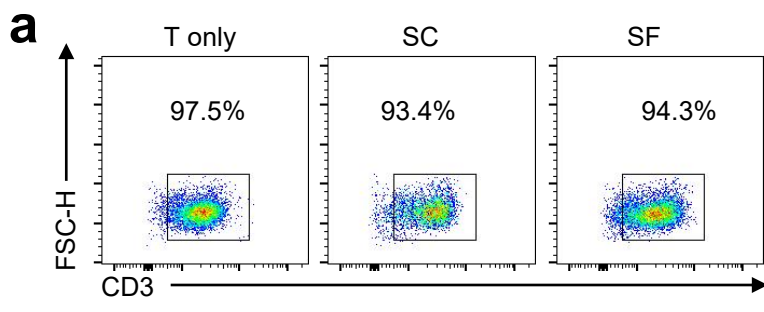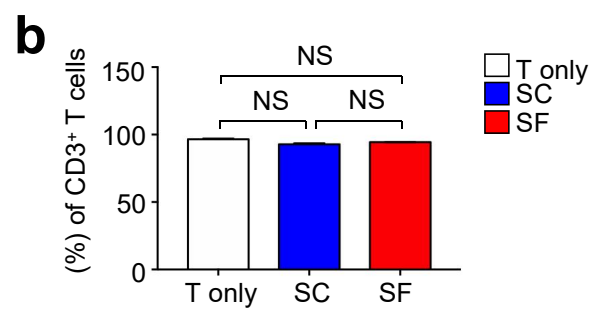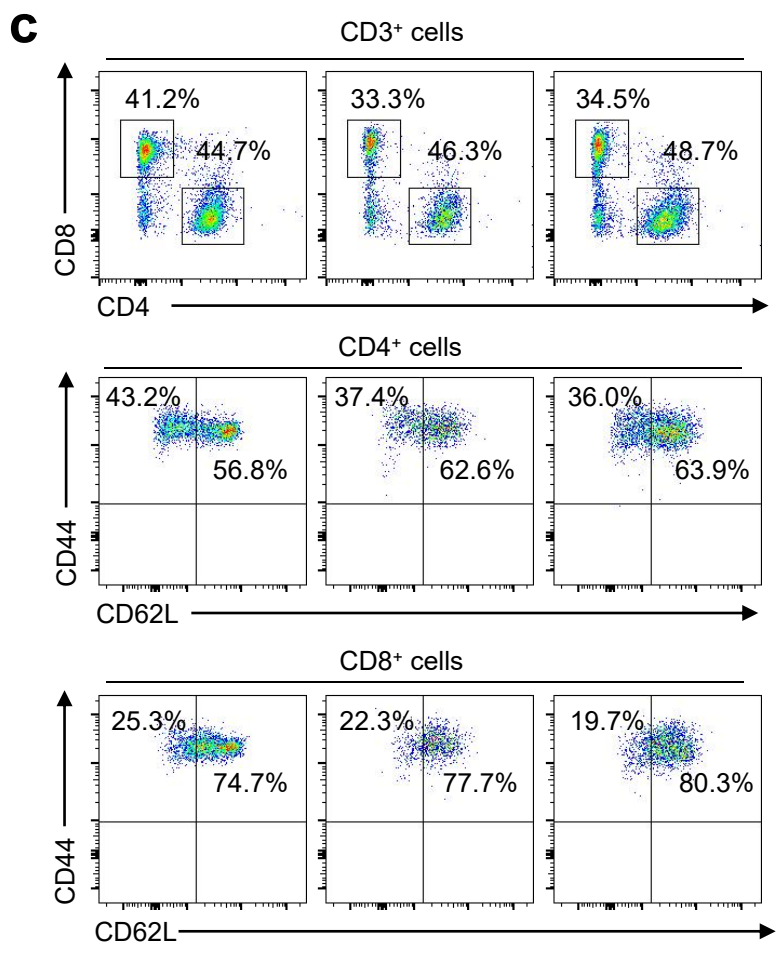

Supplement: Supplementary file 2 — Additional file 2. Figure S2: Comparation of the T cells after coculturing with or without hAMSCs pretreated in SC and SF conditions. [file 13287_2022_3179_MOESM2_ESM.pdf]

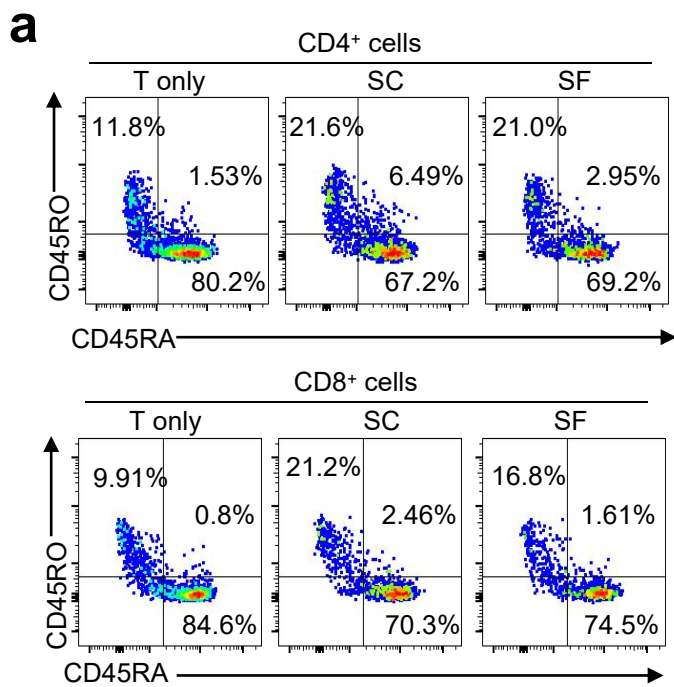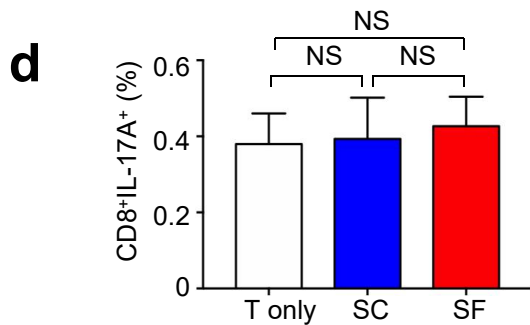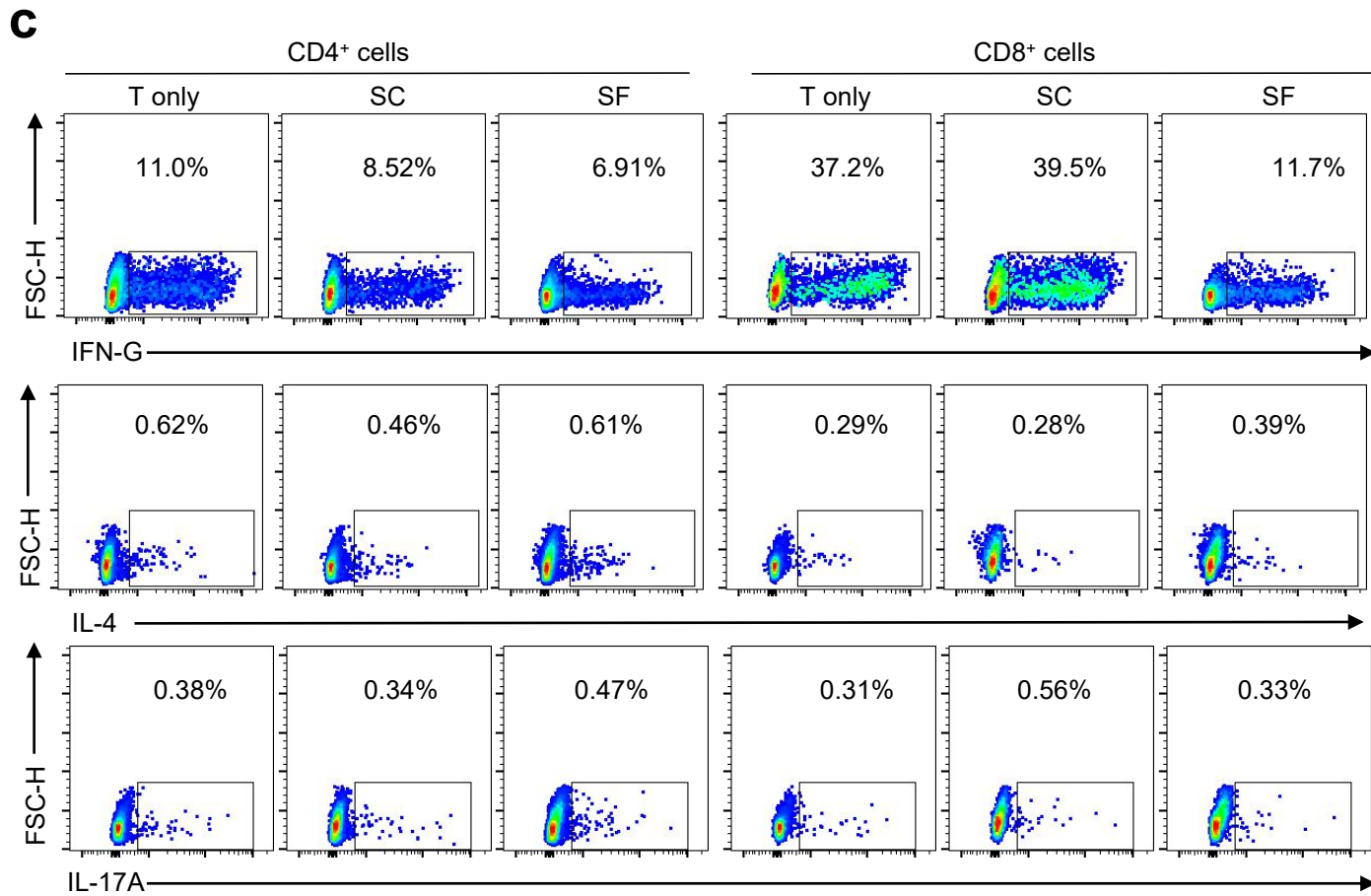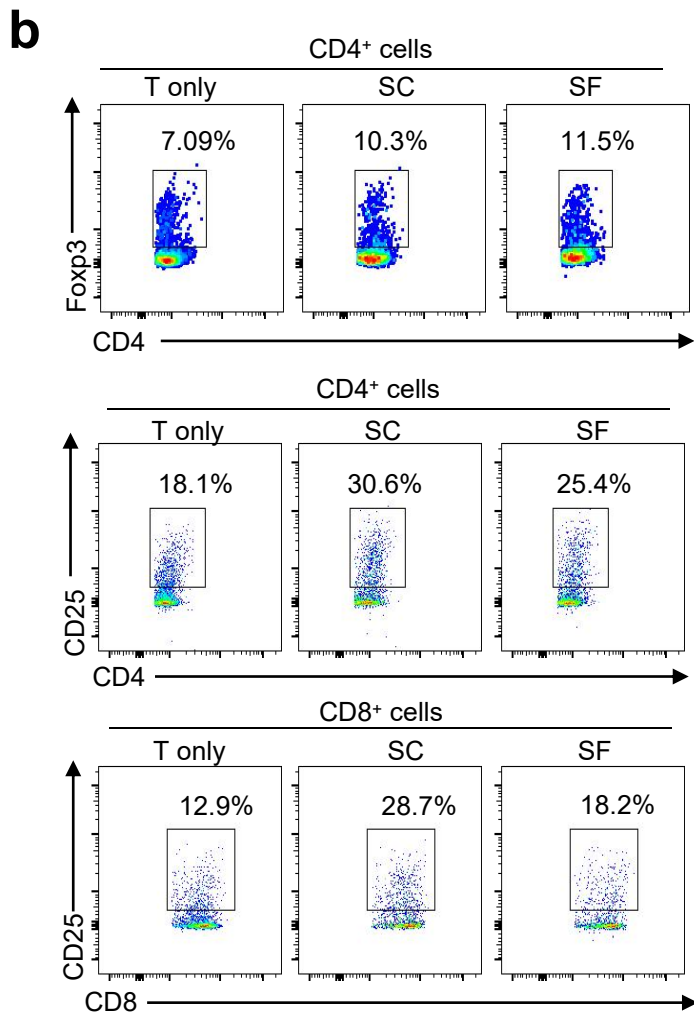

Supplement: Supplementary file 3 — Additional file 3. Figure S3. Comparation of the inhibitory effects of hAMSCs pretreated in SC and SF conditions upon T cells. [file 13287_2022_3179_MOESM3_ESM.pdf]

**a**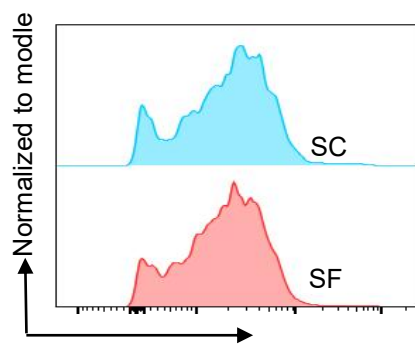**b**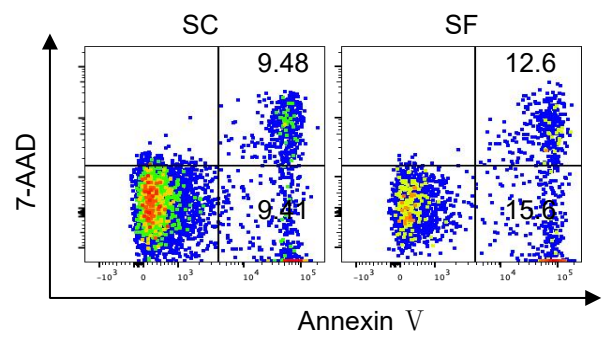

Supplement: Supplementary file 4 — Additional file 4. Figure S4. Cell growth and apoptosis of T lymphocyte subpopulations when cocultured with hAMSCs. [file 13287_2022_3179_MOESM4_ESM.pdf]
