# Supplementary material for: Systematic comparation of the biological and transcriptomic landscapes of human amniotic mesenchymal stem cells under serum-containing and serum-free conditions
Source: Stem Cell Res Ther. 2022 Oct 4;13:490. doi: 10.1186/s13287-022-03179-2 (PMC9530421; doi:10.1186/s13287-022-03179-2)
Supplement: Supplementary file 5 — Additional file 5. The details accompanied with the main manuscript including Additional Figure Legends for Figure S1-S4, Additional Table S1-S3, Additional References were listed. [file 13287_2022_3179_MOESM5_ESM.docx]

## Additional Information

**Systematic Comparation of The Biological and Transcriptomic Landscapes of Human Amniotic Mesenchymal Stem Cells under Serum-containing and Serum-free Conditions**

Yunyan Sun^1,2^, Ti-er Wang^1^, Qianwen Hu^1^, Wenxia Zhang^3^, Yun Zeng^1*^, Xun Lai^2*^, Leisheng Zhang^4-6*^, Mingxia Shi^1*^

**Additional Information**

**Additional Figure Legends for Figure S1-S4**

**Additional Table S1-S3**

**Additional Figure Legends**

**Figure S1 Cell vitality assay of hAMSCs with PI3K-AKT-mTOR signal reactivation and tumorigenicity assay**

**a-b** Representative FCM diagrams **(a)** and statistical analyses **(b)** of the pre-apoptotic or apoptotic property of hAMSCs under SC and SF conditions. **c** Tumorigenicity assay of hAMSCs preconditioned in SC and SF medium for 48 hrs by subcutaneous injection of the 1.0 × 10^7^ hAMSCs mixed with Matrigel into nude mice. Mice with 1.0 × 10^6^ Hela cells mixed with Matrigel injection were used as the positive control. **d** H&E staining was performed for tumor structure detection (scale bar=100 μm). Data were shown as mean±SEM (N=3 independent experiments, n=3 in each group). *, P<0.05; **, P<0.01; NS, not significant.

**Figure S2 Comparation of the T cells after coculturing with or without hAMSCs pretreated in SC and SF conditions**

**a-b** Representative FCM diagrams **(a)** and statistical analysis **(b)** of CD3^+^ T cells after coculturing with or without hAMSCs under SC and SF conditions for 48 hrs. **c** Representative FCM diagrams of CD3^+^CD4^+^ T cells and CD3^+^CD8^+^ T cells (top panel), CD4^+^CD44^+^CD62L^+^ T cells (middle panel) as well as CD8^+^CD44^+^CD62L^+^ T cells (bottom panel) after coculturing with or without hAMSCs under SC and SF conditions for 48 hrs. Data were shown as mean±SEM (N=3 independent experiments). NS, not significant.

**Figure S3 Comparation of the inhibitory effects of hAMSCs pretreated in SC and SF conditions upon T cells**

**a** Representative FCM diagrams of CD3^+^CD45RO^+^ and CD3^+^CD45RA^+^ T cells after coculturing with or without hAMSCs under SC and SF conditions for 48 hrs. **b** Representative FCM diagrams of CD4^+^Foxp3^+^ Treg cells (top panel), CD4^+^CD25^+^ (middle panel) and CD8^+^CD25^+^ T cells (bottom panel) after coculturing with or without hAMSCs under SC and SF conditions for 48 hrs. **c** Representative FCM diagrams of CD4^+^IFN-γ^+^ Th1 cells and CD8^+^IFN-γ^+^ Tc1 cells (top panel), CD4^+^IL-4^+^ Th2 cells and CD8^+^IL-4^+^ Tc2 cells (middle panel) and CD4^+^IL-17A^+^ Th17 cells and CD8^+^IL-17A^+^ T cells (bottom panel) after coculturing with or without hAMSCs under SC and SF conditions for 48 hrs. **d** Statistical analysis of CD8^+^IL-17A^+^ T cells after coculturing with or without hAMSCs under SC and SF conditions for 48 hrs. Data were shown as mean±SEM (N=3 independent experiments). NS, not significant.

**Figure S4. Cell apoptosis of T lymphocyte subpopulations when co-cultured with hAMSCs**

**a-b** Representative FCM diagrams of the cell growth **(a)** and apoptosis **(b)** of the cocultured CD3^+^ T lymphocyte subpopulations with hAMSCs preconditioned in SC and SF conditions for 48 hrs. Data were shown as mean±SEM (N=3). *, P<0.05; **, P<0.01; NS, not significant.

**Additional Table S1 MSCs of different origins**

| **Origins** | **Tissue** | **Abbreviations** | **References** |
| --- | --- | --- | --- |
| Perinatal tissues | Umbilical cord-derived MSCs | UC-MSCs | Ref[1] |
|  | Amniotic membrane-derived MSCs | AMSCs | Ref[2] |
|  | Placenta-derived MSCs | P-MSCs | Ref[3] |
| Adult tissues | Dental pulp of supernumerary teeth-derived MSCs | DPSCs | Ref[4] |
|  | Adipose tissue-derived MSCs | AT-MSCs | Ref[5] |
|  | Bone marrow-derived MSCs | BM-MSCs | Ref[6] |
| Pluripotent stem cells | Embryonic stem cell-derived MSCs | ESC-MSCs | Ref[7, 8] |
|  | Induced pluripotent stem cell-derived MSCs | iPSC-MSCs | Ref[8, 9] |

**Additional Table S2 Antibodies for flow cytometry analysis**

| Antioody | Cat. NO. | Source |
| --- | --- | --- |
| FITC-anti-human-CD90 | 328107 | Biolegend |
| FITC-anti-human-CD3 | 317305 | Biolegend |
| FITC-anti-human-CD45RO | 304204 | Biolegend |
| FITC-anti-human-CD4 | 357405 | Biolegend |
| APC-anti-human-CD105 | 323207 | Biolegend |
| APC-anti-human-CD44 | 397505 | Biolegend |
| APC-anti-human-CD4 | 357407 | Biolegend |
| APC-anti-human-HLA-DR | 327021 | Biolegend |
| APC-anti-human-CD8 | 344721 | Biolegend |
| APC-anti-human-CD25 | 302609 | Biolegend |
| APC-anti-human-IL-4 | 500811 | Biolegend |
| PE-Cy7-anti-human-CD73 | 344009 | Biolegend |
| PE-Cy7-anti-human-CD45 | 304015 | Biolegend |
| PE-Cy7-anti-human-CD34 | 343515 | Biolegend |
| PE-Cy7-anti-human-CD11B | 301321 | Biolegend |
| PE-Cy7-anti-human-CD8 | 344711 | Biolegend |
| PE-Cy7-anti-human-CD45RA | 304125 | Biolegend |
| PE-anti-human-IL-17A | 512305 | Biolegend |
| PE-anti-human-CD44 | 397503 | Biolegend |
| PE-anti-human-Foxp3 | 320107 | Biolegend |
| PE anti-human CD69 Antibody | 310905 | Biolegend |
| APC-Cy7-anti-human-CD4 | 357415 | Biolegend |
| APC-Cy7-anti-human-CD62L | 304813 | Biolegend |
| Percp-cy5.5-anti-human-IFN-G | 502525 | Biolegend |
| APC anti-human Ki-67 Antibody | 350513 | Biolegend |

**Additional Table S3 Primer sequences of candidate genes for qRT-PCR analysis**

| Gene | Forward Primer | Reverse Primer |
| --- | --- | --- |
| *ACTIN* | CTCTTCCAGCCTTCCTTCCT | AGCACTGTGTGTTGGCGTACAG |
| *ADIPOQ* | TGGTCCTAAGGGAGACATCG | TGGAATTTACCAGTGGAGCC |
| *PPAR-γ* | GCTGGCCTCCTTGATGAATA | TGTCTTCAATGGGCTTCACA |
| *RUNX2* | CTCACTACCACACCTACCTG | TCAATATGGTCGCCAAACAGATTC |
| *BGLAP* | GGCGCTACCTGTATCAATGG | TCAGCCAACTCGTCACAGTC |

**Additional References**

1. Zhao Y, Ma J, Yi P, Wu J, Zhao F, Tu W, Liu W, Li T, Deng Y, Hao J *et al*: **Human umbilical cord mesenchymal stem cells restore the ovarian metabolome and rescue premature ovarian insufficiency in mice**. *Stem Cell Res Ther* 2020, **11**(1):466.

2. Dalouchi F, Falak R, Bakhshesh M, Sharifiaghdam Z, Azizi Y, Aboutaleb N: **Human amniotic membrane mesenchymal stem cell-conditioned medium reduces inflammatory factors and fibrosis in ovalbumin-induced asthma in mice**. *Exp Physiol* 2021, **106**(2):544-554.

3. Hou H, Zhang L, Duan L, Liu Y, Han Z, Li Z, Cao X: **Spatio-Temporal Metabolokinetics and Efficacy of Human Placenta-Derived Mesenchymal Stem/Stromal Cells on Mice with Refractory Crohn's-like Enterocutaneous Fistula**. *Stem Cell Rev Rep* 2020, **16**(6):1292-1304.

4. Yao J, Chen N, Wang X, Zhang L, Huo J, Chi Y, Li Z, Han Z: **Human Supernumerary Teeth-Derived Apical Papillary Stem Cells Possess Preferable Characteristics and Efficacy on Hepatic Fibrosis in Mice**. *Stem Cells Int* 2020, **2020**:6489396.

5. Chen S, Cui G, Peng C, Lavin MF, Sun X, Zhang E, Yang Y, Guan Y, Du Z, Shao H: **Transplantation of adipose-derived mesenchymal stem cells attenuates pulmonary fibrosis of silicosis via anti-inflammatory and anti-apoptosis effects in rats**. *Stem Cell Res Ther* 2018, **9**(1):110.

6. Friedenstein AJ, Petrakova KV, Kurolesova AI, Frolova GP: **Heterotopic of bone marrow. Analysis of precursor cells for osteogenic and hematopoietic tissues**. *Transplantation* 1968, **6**(2):230-247.

7. Wei Y, Hou H, Zhang L, Zhao N, Li C, Huo J, Liu Y, Zhang W, Li Z, Liu D *et al*: **JNKi- and DAC-programmed mesenchymal stem/stromal cells from hESCs facilitate hematopoiesis and alleviate hind limb ischemia**. *Stem Cell Res Ther* 2019, **10**(1):186.

8. Zhang L, Wang H, Liu C, Wu Q, Su P, Wu D, Guo J, Zhou W, Xu Y, Shi L *et al*: **MSX2 Initiates and Accelerates Mesenchymal Stem/Stromal Cell Specification of hPSCs by Regulating TWIST1 and PRAME**. *Stem Cell Reports* 2018, **11**(2):497-513.

9. Zhang L, Wei Y, Chi Y, Liu D, Yang S, Han Z, Li Z: **Two-step generation of mesenchymal stem/stromal cells from human pluripotent stem cells with reinforced efficacy upon osteoarthritis rabbits by HA hydrogel**. *Cell Biosci* 2021, **11**(1):6.
